# An fMRI analysis of verbal and non-verbal working memory in people with a past history of opioid dependence

**DOI:** 10.3389/fnins.2023.1053500

**Published:** 2023-04-05

**Authors:** Joshua G. Berenbaum, Prianca A. Nadkarni, Cherie L. Marvel

**Affiliations:** Department of Neurology, Johns Hopkins University School of Medicine, Baltimore, MD, United States

**Keywords:** opioids, heroin, cognition, neuroimaging, methadone, substance use, brain, addiction

## Abstract

**Introduction:**

Working memory describes the ability to maintain and manipulate information held in mind, and it is a fundamental aspect of executive function. Within drug addiction, impairments of executive control over behavior are thought to lead to poor decision making and risky behaviors. Previous research has demonstrated working memory (WM) and executive function difficulties in opioid-dependent individuals, but the neural underpinnings of such impairments in this population are not well understood.

**Methods:**

This study used functional magnetic resonance imaging to examine the neural mechanisms involved in WM in 13 opioid-dependent, methadone-maintained participants (OP) and 13 matched, healthy controls (HC). A Sternberg item-recognition task was administered with three conditions: (1) a “verbal” condition in which participants determined whether any six visually presented target letters matched a probe item that was presented 4–6 s later, (2) a “non-verbal” condition in which participants were presented with a Chinese character and, following a 4–6 s delay, determined whether the character matched the probe item, and (3) a “control” condition in which participants were presented with three horizontal lines and following the same delay, determined whether the lines matched a probe item (always the same three lines). Functional magnetic resonance imaging (fMRI) contrasts focused on the delay (or “maintenance”) phase for verbal and non-verbal conditions relative to the control condition.

**Results:**

Accuracy on the WM task did not differ between groups, but the OP group was significantly slower to respond. The fMRI imaging results indicated differences in brain activity between the OP and HC groups. fMRI-guided regions of interest correlated with age of first alcohol and THC use, suggesting that early substance use, in addition to years of opioid-abuse, may have played a role in the OP group’s WM performance.

**Discussion:**

A deeper understanding of these neural differences between opioid-dependent individuals and their healthy control counterparts helps shed light on fundamental ways in which substance use impacts the brain and cognition, potentially opening up novel avenues for therapeutic targets to treat substance use disorder.

## 1. Introduction

Working memory (WM) refers to the ability to mentally maintain and manipulate information for brief periods of time (Baddeley, 1992). This information is essential for completing the complex cognitive tasks of life, such as driving, talking, writing, and problem solving. WM is also extremely important for executive functioning, logical thinking and reasoning (Lezak et al., 2004; Mandler, 2007). As a result, substance-abuse populations with cognitive impairments (e.g., alcoholics, cocaine-users, and opioid-users) in WM are of much interest, as the WM impairment not only causes these individuals to live hindered lives (Grant, 1987; Darke et al., 2000; Specka et al., 2000; Davis et al., 2002; Sullivan et al., 2010; Soar et al., 2012) but also acts as a risk factor for further substance abuse and poor decision making (Finn, 2002; Squeglia et al., 2012). Therefore, it is important to understand how WM may be disrupted in these populations. Considering the increasing prevalence of heroin and opioid abuse, opioid-dependent users are an important focus in studying cognitive impairments (Rass et al., 2014).

A popular model for WM proposed by Baddeley and Hitch (1974) consists of three components: a central executive involved in allocating attentional resources, a phonological loop that supervises verbalizable content, and a visuospatial sketch pad that manages visual (and often non-verbalizable) content. The phonological loop can be further divided into a phonological store, temporarily holding memory traces, and an articulatory rehearsal process, utilizing internal speech to refresh the information (Baddeley, 2003; Ackermann et al., 2004). This active rehearsal with internal speech consists of mentally vocalizing the verbalizable content (words and numbers) but not overtly speaking it out loud, thus engaging a speech-related motor network. Verbal WM functions through the phonological loop and utilizes a neural circuit involving secondary (rather than primary) motor regions, such as the inferior frontal and dorsolateral prefrontal brain regions, premotor and supplementary motor areas (SMAs), the inferior parietal lobe (IPL), and the cerebellum (for a review, see Marvel et al., 2019). Furthermore, verbal WM shares functional overlap with overt and covert speech, such as in Broca’s area, demonstrating involvement in a language-related cerebro-cerebellar circuit and reflecting pre-movement processes that occur prior to overt motor execution (speech in this case) (Marvel and Desmond, 2010; Friederici, 2011; Marvel et al., 2019; Yan et al., 2021).

On the other hand, non-verbal WM pertains to non-verbalizable content like symbols or written characters unknown to the individual (e.g., Chinese characters viewed by someone who cannot read or write the language). Non-verbal WM rehearsal might consist of mental drawing or tracing of the content, and similar to verbal WM, it also utilizes secondary motor regions, such as the premotor cortex, SMA, and superior cerebellum (Pollmann and Von Cramon, 2000; Champod and Petrides, 2007; Liao et al., 2014; Sobczak-Edmans et al., 2016; Marvel et al., 2019). This overlap was demonstrated in a study by Liao et al. (2014), in which transcranial magnetic stimulation (TMS) was applied to participants’ primary motor cortices, in order to propagate activation throughout the entire motor network, including secondary motor regions, during WM rehearsal of verbal and non-verbal information. TMS disrupted WM performance for both types of stimuli, neither of which was affected by sham TMS or TMS to the visual cortex. Moreover, the degree of TMS disruption was proportionate to the magnitude of self-reported use of motor-related rehearsal strategy. Taken together, these findings suggest at least partial overlap of an underlying motor circuit that supports verbal and non-verbal WM.

The Sternberg task is a common paradigm used to assess WM. It consists of three phases: encoding, maintenance, and retrieval (Sternberg, 1966). In this paradigm, novel stimuli are presented to the participant during a brief period (i.e., seconds), and the participant is instructed to study the information (encoding). The stimuli are removed from view (if visual), and participants hold the information in mind (maintenance). Typically, participants are instructed to refrain from using overt strategies, such as speaking aloud, to rehearse the content. Finally, a probe item is presented to the participant, and they decide whether the probe matches the initial stimuli presented in the encoding phase (retrieval).

Applying this type of paradigm in conjunction with functional magnetic resonance imaging (fMRI) methods, it has been reported that healthy individuals increase cerebro-cerebellar activity in association with increasing cognitive load (e.g., increasing number of items to encode) (Desmond et al., 1997; Chen and Desmond, 2005; Marvel and Desmond, 2012). Opioid-dependent individuals have been shown to experience a similar pattern of hyperactivity along with other brain regions, such as the inferior frontal gyrus and precuneus, but to a greater spatial extent and in response to a lower cognitive load relative to that of healthy individuals (Marvel et al., 2012; Sadananda et al., 2021). Relatedly, neuroimaging studies have also revealed structural brain abnormalities in people with opioid dependence. For instance, reductions in gray matter volume have been reported in the right prefrontal cortex, left SMA, bilateral cingulate cortices (CC), basal ganglia, and insula for opioid-dependent subjects (Liu et al., 2009; Tolomeo et al., 2016, 2018; Bach et al., 2021; Monick et al., 2022). Reductions in gray matter have been reported in the prefrontal and temporal cortices of methadone maintenance patients (Lyoo et al., 2006). Furthermore, the magnitudes of overall gray matter density reduction and resting-state functional connectivity between the prefrontal cortex, insula, basal ganglia, and cerebellum in opioid-dependent individuals have been reported as negatively correlated with duration of opioid use (Upadhyay et al., 2010; Yuan et al., 2010a,b; Monick et al., 2022).

In line with these structural and functional changes, WM impairments have repeatedly been demonstrated in studies of individuals with opioid dependence. For example, Mitrovic et al. (2011) and Wang et al. (2008) both found an impairment in verbal WM for opioid-dependent individuals. Mitrovic et al. (2011) utilized the Rey Auditory Verbal Learning Test (RAVLT) (Rey, 1964) in which participants were presented with 15 words lists and were asked to recall as many as they could immediately afterward. Participants with a history of heroin abuse longer than 1 year scored significantly lower on immediate word recall than did healthy controls (HC), and the longer their history of heroin abuse, the lower their ability to recall the words. Wang et al. (2008) utilized two verbal WM tasks: an N-back task in which participants recalled a letter “n” digits prior in an orally presented list, and a digit span task in which participants repeated a sequence of letters forward or backward. The participants with a history of heroin abuse performed normally on the digit span task but demonstrated impaired verbal WM on the N-back task compared to HC. Additionally, Ersche et al. (2006) and Tolomeo et al. (2019) found visuospatial memory deficits in opioid-dependent participants, implicating a non-verbal WM impairment as well. These studies included the pattern recognition memory task from the Cambridge Neuropsychological Test Automated Battery (CANTAB) (Fray et al., 1996), which involved participants viewing sets of geometric patterns and later choosing the pattern they recognized from a two-alternative forced choice test of the patterns. Participants with a history of heroin abuse consistently demonstrated lower performance than did HC.

While the verbal and non-verbal WM impairments stemming from opioid abuse have been well-demonstrated, there is still a knowledge gap as to the physiological brain processes by which this WM deficit functions in the opioid-dependent population. WM is essential for daily life and an impairment can be a severe risk factor for further substance abuse and other harmful behaviors. Therefore, the present study used event-related fMRI to examine the neural correlates of verbal and non-verbal WM in people with a past history of opioid dependence who were currently undergoing methadone treatment. It was hypothesized that the opioid-dependent participants would express greater neural activity than HC in several brain regions observed in previous research, such as cerebro-cerebellar, SMA, and inferior frontal gyrus hyperactivity, as compensation for their cognitive impairments on the WM task.

## 2. Materials and methods

### 2.1. Participants

Thirteen opioid-dependent, methadone-maintained individuals (OP; eight males) and 13 HC (eight males) without histories of drug dependence were enrolled in the study. OP participants were recruited from the Johns Hopkins Behavioral Pharmacology Research Unit (BPRU), and controls were recruited from the Baltimore community. All participants were native English speakers, right-handed, and had no known neurological conditions or history of head trauma. Because one condition of the WM tasks involved the use of Chinese characters, all participants were screened for reading and writing knowledge in Mandarin, Cantonese, or Japanese (all of which share script features), and they were excluded if so. All participants were administered an abbreviated Structured Clinical Interview for DSM-IV Axis I (SCID), which established a history of heroin and/or other drug dependence in the OP group (and lack thereof in the control group), determined the presence of alcohol dependence (exclusionary for both groups), and determined a history of Axis I disorders related to mood or psychosis that were unrelated to substance use (exclusionary for both groups). All demographic information and performance on several cognitive and motor tasks can be seen in Table 1. These tasks included the Barratt Impulsivity Test (Patton et al., 1995), the Digit Span memory test (Wechsler, 2008), a finger tapping task (Reitan and Wolfson, 1993), a color change visual WM test [Luck and Vogel, 1997, as described in Anderson et al. (2013) and Anderson et al. (2016)], and the Clinical Opiate Withdrawal Scale (COWS) (Wesson and Ling, 2003).

**TABLE 1:** Demographic variables of the study groups.

Demographic	OP (*n* = 13)	HC (*n* = 13)	Group differences *p*-Value
Gender, M:F	8:5	8:5	–
Age (years)	43.2 (11.0) [36.7, 50.3], *W* = 0.91, *p* = 0.18	43.5 (11.3) [36.5, 49.8], *W* = 0.89, *p* = 0.08	*p* = 0.943
Education (years)*	11.9 (1.67) [10.9, 12.9], *W* = 0.93, *p* = 0.31	14.5 (1.61) [13.5, 15.4], *W* = 0.82, ***p* = 0.01**	***p* = 0.002**
Race (White:Black:Asian)	7:6:0	7:4:2	*p* = 0.30
Household income ($ monthly)	2,330 (2832) [304, 4,356], *W* = 0.65, ***p* < 0.001**, *n* = 10	10,995 (30,212) [–9,302, 31,292], *W* = 0.38, ***p* < 0.001**, *n* = 11	*p* = 0.646
Methadone dosage (mg)	85.3 (32.8) [range = 14 – 140 mg] [64.2, 109], *W* = 0.95, *p* = 0.65	N/A	–
BIS*	63.0 (11.3) [56.2, 69.8], *W* = 0.96, *p* = 0.69	53.6 (7.53) [49.1, 58.2], *W* = 0.94, *p* = 0.45	***p* = 0.021**
Digit span sum*	14.2 (2.97) [12.4, 16.0], *W* = 0.98, *p* = 0.96	17.1 (3.97) [14.7, 19.5], *W* = 0.97, *p* = 0.88	***p* = 0.045**
Right hand finger tapping score	44.0 (9.46) [38.3, 50.0], *W* = 0.89, *p* = 0.11	49.0 (11.5) [42.1, 56.0], *W* = 0.62, ***p* < 0.001**	*p* = 0.522
Color change visual WM score*	1.37 (0.51) [1.06, 1.67], *W* = 0.90, *p* = 0.14	2.00 (0.57) [1.66, 2.34], *W* = 0.98, *p* = 0.98	***p* = 0.006**
COWS score	1.73 (2.28) [0.19, 3.26], *W* = 0.76, ***p* = 0.003**, *n* = 11	N/A	–

OP, opioid dependence history; HC, healthy controls; M, male; F, female; BIS, Barratt Impulsiveness Scale; N/A, not applicable indicates that no participants in this group had data for the category. Ratios are presented for categorical variables; mean (SD), 95% confidence interval [lower limit, upper limit] are presented for continuous variables; Shapiro–Wilk tests were used for tests of normality. Significant values of the Shapiro–Wilk tests are denoted in bold, *p* < 0.05, two-tailed. When Shapiro–Wilk tests were significant, non-parametric tests were subsequently used for group comparisons.

*Group differences, *p* < 0.05.

Opioid-dependent participants had been receiving methadone treatment for at least 10 months and were on a stable dose (within 5% of current dose) for at least 2 months prior to the MRI. All opioid-dependent participants were abstinent at the time of testing, which was confirmed in the OP group by: (1) regular (at least bi-monthly) urinalysis taken as part of their treatment within the BPRU, (2) counselor-provided information, and (3) participant-provided information. In controls, drug abstinence was based on information provided by the participant. In addition, both groups were screened for recent drug and alcohol use via urinalysis and breathalyzer on the day of the initial eligibility screening and again on the day of the MRI (conducted within 2 weeks of the initial eligibility screening). Women who may become pregnant were given a urine pregnancy test prior to entering the MRI environment. The drug screen (Aim Screen Multidrug 9 by Germaine Laboratories) tested for the presence of the following substances in the urine: cocaine, amphetamine, methamphetamine, marijuana, methadone, opiates, phencyclidine, barbiturates, and benzodiazepines. A detailed history of lifetime drug and alcohol exposure was obtained in both groups using a modified version of the Lifetime Drug Use Questionnaire (Czermak et al., 2005; Marvel et al., 2012; Anderson et al., 2013, 2016; Monick et al., 2022). These measures can be seen in Table 2, including age of first substance use, duration of use (time between the first and last substance use), abstinence duration since last substance use, and for some substances, the calculated average frequency of use during periods of use, as modeled after the calculation in Monick et al. (2022). This measure for frequency of use further characterized the cocaine and opioid use in participants, providing a measure that took into consideration the changing frequencies with which participants used substances throughout their total duration of use. For instance, participants might have used heroin at least once weekly during one period in their life, but multiple times daily at a different time. A rating from 1 (once annually) to 5 (multiple times a week) was taken for each period of use, and the ratings were averaged in accordance with the duration of the phases to provide the reported frequency values. It is worth noting that the methadone use reported in Table 2 refers to periods of methadone abuse, as opposed to the methadone treatment utilized at the time of the study. Opioid-dependent participants refrained from taking their morning dose of medication the day of the MRI to prevent the acute sedating effects of methadone from impacting study performance. The time frame of 20–24 h *post* last methadone dose is well past methadone’s peak effects on cognition and physiology, and represents a “trough” period when methadone’s effects return to baseline (Walsh et al., 1994; Eissenberg et al., 1999). Subjects were not expected to be in withdrawal, but the COWS was completed following the MRI scan (Wesson and Ling, 2003; Tompkins et al., 2009).

**TABLE 2:** Drug history by group.

	Age of first use (years)		Duration of use (years)		Abstinence (years)		Frequency of use	
	**OP**	**HC**	**OP**	**HC**	**OP**	**HC**	**OP**	**HC**
Alcohol	12.9* (2.63) [11.2, 14.7], *W* = 0.94, *p* = 0.57, *n* = 11	15.8 (2.44) [14.3, 17.4], *W* = 0.95, *p* = 0.67, *n* = 12	27.1 (12.4) [18.8, 35.4], *W* = 0.90, *p* = 0.19, *n* = 11	25.2 (14.9) [15.7, 34.6], *W* = 0.83, ***p* = 0.02**, *n* = 12	4.26* (6.49) [−0.09, 8.62], *W* = 0.67, ***p* < 0.001**, *n* = 11	2.90 (8.51) [−2.51, 8.30], *W* = 0.36, ***p* < 0.001**, *n* = 12	–	–
Nicotine	12.9 (2.58) [11.3, 14.6], *W* = 0.78, ***p* = 0.006**, *n* = 12	15.2 (5.02) [11.4, 19.1], *W* = 0.91, *p* = 0.30, *n* = 9	23.0^∧^ (13.8) [14.2, 31.8], *W* = 0.95, *p* = 0.63, *n* = 12	11.0 (13.2) [0.89, 21.1], *W* = 0.78, ***p* = 0.01**, *n* = 9	5.95 (11.4) [−1.28, 13.2], *W* = 0.59, ***p* < 0.001**, *n* = 12	16.7 (19.4) [1.78, 31.6], *W* = 0.74, ***p* = 0.004**, *n* = 9	–	–
Tetrahydrocannabinol (THC, or cannabis)	15.0^∧^ (3.22) [13.1, 16.9], *W* = 0.80, ***p* = 0.006**, *n* = 13	16.8 (2.93) [13.8, 19.9], *W* = 0.89, *p* = 0.29, *n* = 6	18.3* (13.4) [10.2, 26.4], *W* = 0.94, *p* = 0.40, *n* = 13	6.00 (5.62) [0.10, 11.9], *W* = 0.88, *p* = 0.27, *n* = 6	9.85 (10.4) [3.54, 16.2], *W* = 0.81, ***p* = 0.009**, *n* = 13	20.0 (16.1) [3.11, 36.8], *W* = 0.81, *p* = 0.08, *n* = 6	–	–
Cocaine	22.3 (6.74) [17.8, 26.8], *W* = 0.80, ***p* = 0.008**, *n* = 11	22.0 (7.07) [−41.5, 85.5], *n* = 2	15.0 (11.5) [7.25, 22.8], *W* = 0.87, *p* = 0.07, *n* = 11	7.50 (6.36) [−49.7, 64.7], *n* = 2	5.76 (8.10) [0.33, 11.2], *W* = 0.67, ***p* < 0.001**, *n* = 11	25.0 (3.43) [−5.79, 55.8], *n* = 2	3.52 (0.51) [3.18, 3.86], *W* = 0.83, ***p* = 0.03**, *n* = 11	1.00 (0.00) [1.00, 1.00], *n* = 2
Heroin	24.3 (7.79) [19.6, 29.0], *W* = 0.88, *p* = 0.08, *n* = 13	N/A	15.8 (11.0) [9.12, 22.4], *W* = 0.90, *p* = 0.14 *n* = 13	N/A	3.08 (2.54) [1.54, 4.62], *W* = 0.85, ***p* = 0.03**, *n* = 13	N/A	3.98 (0.43) [3.72, 4.24], *W* = 0.92, *p* = 0.21, *n* = 13	N/A
Oxycontin	20.4 (7.30) [11.3, 29.5], *W* = 0.78, *p* = 0.06, *n* = 5	N/A	9.20 (5.36) [2.54, 15.9], *W* = 0.78, *p* = 0.06, *n* = 5	N/A	2.97 (2.48) [−0.11, 6.04], *W* = 0.86, *p* = 0.23, *n* = 5	N/A	3.87 (1.12) [2.48, 5.26], *W* = 0.86, *p* = 0.22, *n* = 5	N/A
Methadone	26.0 (10.1) [13.5, 38.5], *W* = 0.96, *p* = 0.82, *n* = 5	N/A	3.20 (3.03) [−0.57, 6.97], *W* = 0.73, ***p* = 0.02**, *n* = 5	N/A	8.42 (9.31) [−3.14, 20.0], *W* = 0.83, *p* = 0.13, *n* = 5	N/A	3.20 (0.447) [2.64, 3.76], *W* = 0.55, ***p* < 0.001**, *n* = 5	N/A

Values and statistics are reported as in Table 1. N/A, not applicable indicates that no participants in this group had data for the category. Group differences were not run for the cocaine variable due to a low n for the HC group. Note that methadone is listed within the context of a drug of abuse rather than treatment.

*Group differences, *p* < 0.05, ^∧^group differences, *p* < 0.10.

Originally, the study included 15 OP and 16 HC participants. One OP participant was excluded from the dataset because there was an incidental finding on the MRI. A second OP participant was excluded because of excessive alcohol consumption that had not met criteria for alcohol dependence during the SCID, but the self-reported amount and extent of use was excessively high and may have interfered with the interpretation of results, especially impacting the cerebellum where alcohol is known to have deleterious effects (Luo, 2015). Three HC participants were subsequently removed from the dataset to include those HC that best matched the 13 remaining OP participants in terms of age, gender, education and, thereby, provided equal sample sizes per group.

This research was approved by the Johns Hopkins Institutional Review Board and in accordance with the Helsinki Declaration of 1975. All participants gave their written informed consent and were paid for their participation.

### 2.2. Statistical analysis of behavioral variables

The demographic and behavioral data collected in this study contained continuous (e.g., age, education, and digit span), categorical (e.g., gender and race), and ordinal variables (e.g., COWS score). Shapiro–Wilk tests were used to determine if the continuous variables (such as participant age) followed a normal distribution. If a variable was non-normal, Mann–Whitney U tests were used to compare group scores, and Spearman’s correlations were conducted to associate variables. Otherwise, independent-samples *t*-tests and Pearson’s correlations were conducted. Mixed-design ANOVAs were used to compare repeated measures between the OP and HC groups (e.g., mean accuracy and reaction time across conditions) (Mauchly’s Test of Sphericity was first used to determine if the ANOVAs violated the sphericity assumption; if so, degrees of freedom were corrected using Greenhouse–Geisser estimates of sphericity). *Post hoc* pairwise comparisons were determined using Least Significant Difference tests (adjusted for multiple comparisons), which identified the populations or conditions when means were significantly different. All tests were two-tailed, with an alpha level < 0.05 to define statistical significance. Statistics were performed using IBM SPSS Statistics, Macintosh, version 27.0 (IBM Corp., Armonk, NY, USA).

### 2.3. Functional MRI WM task

**FIGURE 1:**
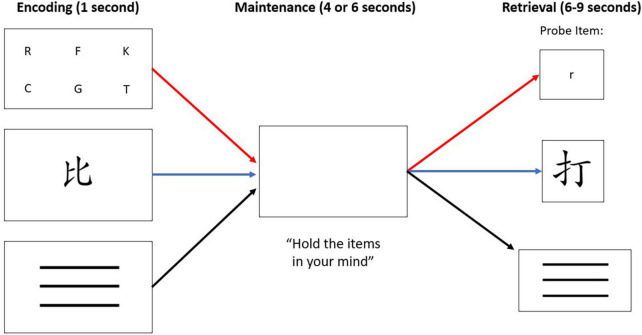
The WM paradigm consisted of three conditions: verbal stimuli (letters), non-verbal stimuli (Chinese character), or “control condition” stimuli that was represented with three horizontal lines. Each trial, regardless of condition, consisted of three phases: encoding, maintenance, and retrieval. The red path depicts the verbal condition, the blue path depicts the non-verbal condition, and the black path depicts the control condition. Note that a match-to-target is depicted in the verbal condition. A non-match-to-target is depicted in the non-verbal condition. All control condition trials were a match-to-target, minimizing the WM load demand in that condition.

Participants performed a WM task, as in Figure 1. The WM task contained three conditions: (1) a verbal condition in which participants studied and matched target letters with a probe item, (2) a non-verbal condition in which participants (who could not read or write Mandarin, Cantonese, or Japanese) were presented with a Chinese character and identified whether it matched a probe item, and (3) a control condition where participants were presented with a simple line pattern and identified whether it matched a probe item. Participants were visually presented with verbal, non-verbal, or control target cues during an encoding phase, followed by a brief maintenance phase to silently rehearse the targets while viewing a blank white screen. During the retrieval phase, subjects were presented with a probe item and instructed to respond via button press to indicate whether the probe matched the target. The targets in the verbal condition consisted of six uppercase English letters, in the non-verbal condition consisted of one Chinese character, and in the control condition consisted of three horizontal lines. The Chinese character was considered to be non-verbal in this study because the participants were native English speakers who did not read Mandarin, Cantonese, or Japanese and could not easily apply verbal cues to the characters. In the retrieval phase, participants used their right hand to press button 1 with their index finger to indicate a match, or button 2 with their middle finger to indicate a non-match, of the probe to the encoded target. On control trials, all probes matched the target. They were instructed to not speak aloud while rehearsing the information and to respond as quickly and accurately as possible once the probe was presented.

The three conditions were co-mingled within two separate MRI runs of 40 trials each, the order of which was counterbalanced across participants. Within a run, trials were jittered with an inter-trial interval (ITI) that lasted 6–9 s. A trial was initiated at the end of the ITI rather than triggered by user response. Ten trials (25%) per run did not include any probe at all so that the hemodynamic response measured during the maintenance phase could be visualized more clearly, without interference from the button press of the retrieval phase, as done in previous studies (Marvel and Desmond, 2012; Marvel et al., 2012, 2022). In the no-probe trials, participants viewed a blank screen throughout the retrieval phase, and no response was expected. The number of trials for each condition, maintenance duration (4 or 6 s), expected response (match or non-match, except for control trials), and ITI duration (6, 7, 8, or 9 s) were equated and pseudo-randomized such that the presentation of identical parameters was limited to three consecutive trials. Participants were trained on the rules of the task on 10 trials prior to entering the MRI environment. The behavioral variables of interest were accuracy and response times (RT) on each trial.

### 2.4. Functional MRI acquisition and analysis

MRI data were collected on a Philips 3T Intera scanner and 8-channel head coil. Task stimuli were designed using E-prime Professional 1.1 software (E-Prime, 2002) on a Hewlett Packard xw4300 workstation running Windows XP Pro. Visual stimuli were rear-projected onto a screen behind the participant’s head and reflected onto a mirror directly within the participant’s line of sight, just beyond the head coil. The structural MRI protocol consisted of a T1-weighted MPRAGE (TR = 6.83 ms; TE = 3.22 ms; flip = 8°, in-plane resolution = 0.75 mm; slice thickness = 1 mm skip 0 mm; 170 sagittal slices; FOV = 240 mm). fMRI data were collected using a T2*-weighted gradient echo EPI pulse sequence (TR = 1,000 ms; TE = 30 ms; flip = 61°; in-plane resolution = 2.75 mm; slice thickness = 6 mm skip 1 mm; 19 oblique-axial slices; FOV = 220 mm). T2*-weighted images were acquired in the oblique-axial plane rotated 25°clockwise with respect to the AC-PC line in order to optimize imaging of the cerebellum and neocortex. The start of the fMRI scan was triggered by E-prime software at the beginning of each run.

To perform whole-brain analyses, standard image preprocessing steps were performed using SPM12 (Wellcome Department of Cognitive Neurology), including slice timing correction (reference = middle slice) and motion correction. Structural scans were co-registered to the mean image, normalized to the Montreal Neurological Institute (MNI) stereotaxic space, and underwent spatial smoothing (FWHM = 8 mm). Individual hemodynamic response functions (HRFs) were obtained using an event-related finger tapping paradigm that was collected during the same fMRI session to create regressors derived from the peak activation voxel within the left primary motor cortex, following Chen and Desmond (2005), Desmond et al. (1997), Marvel and Desmond (2012), and Marvel et al. (2022). Individual HRFs were obtained instead of using canonical HRFs in the analyses to control for potential individual (or clinical group) HRF differences (Ances et al., 2011; Kim and Ogawa, 2012) and had been processed previously using SPM8. Individualized regressors were convolved with reference waveforms from the maintenance phase of the task on correct trials only (event-related). Incorrect trials were excluded from the regressor and treated as a nuisance variable. Statistical whole-brain maps were computed using the general linear model approach, with a high pass filtering of 128 s. Random effects analyses were used to create a statistical map of the blood oxygen level dependence (BOLD) signal for each condition. This was performed by computing a contrast volume per participant and using the volumes to compute a one-sample *t*-test at every voxel that represented the BOLD signal difference between the target condition (verbal or non-verbal) minus the control condition. Verbal – control condition and non-verbal – control condition contrasts were examined within OP and HC groups separately. Between-group analyses involved a double subtraction method that first included the initial contrast between conditions within each group, then subtracting this contrast between the two groups.

### 2.5. Region of interest analyses

Using the MarsBaR toolbox for SPM12, functionally defined regions of interest (ROIs) were created from activation clusters identified in whole-brain, within-groups contrasts for verbal and non-verbal conditions. ROIs were thresholded for cluster significance at *p* < 0.001, with an uncorrected cluster significance of *p* < 0.05 and a minimum of 10 voxels per cluster. For clusters >1,000 voxels, the threshold for significance was lowered to *p* < 0.00001 to create a manageable cluster size for the ROI analyses. MRI signals within the ROIs were obtained for every participant to be used for correlation analyses involving behavioral task performance variables, such as task accuracy and RTs for accurate trials, and drug history variables.

## 3. Results

### 3.1. Demographics

Demographic information for both groups can be seen in Table 1. Groups were equated for age in years, [OP, *M* = 43.2 (SD = 11.0); HC, *M* = 43.5 (SD = 11.3); *t*(24) = 0.073, *p* = 0.943, *d* = 0.029]. Education level, however, was significantly lower in the OP group (Mdn = 12.0) than in the HC group (Mdn = 15.0), *U* = 24.0, *p* = 0.002, *r* = 0.62. The OP group scored higher than the HC group did on the Barratt Impulsiveness Scale (BIS) [OP, *M* = 63.0 (SD = 11.3); HC, *M* = 53.6 (SD = 7.53); *t(*24) = −2.49, *p* = 0.021, *d* = −0.98]. The OP group scored lower than the HC group did on the digit span test [OP, *M* = 14.2 (SD = 2.97); HC, *M* = 17.1 (SD = 3.97); *t*(24) = 2.13, *p* = 0.045, *d* = 0.83] and on the color change visual WM task [OP, *M* = 1.37 (SD = 0.51), HC, *M* = 2.00 (SD = 0.57); *t*(24) = 2.98, *p* = 0.006, *d* = 1.18].

### 3.2. Drug history

Drug history values of both groups are reported in Table 2. All opioid-dependent participants had abstained from illicit drug use for 204–892 days prior to testing. An independent-samples *t*-test indicated that there was a significantly lower age of first alcohol use in the OP group than in the HC group [OP, *M* = 12.9 (SD = 2.63); HC, *M* = 15.8 (SD = 2.44); *t*(21) = 2.76, *p* = 0.012, *d* = 1.12]; meanwhile, the OP group reported a longer duration of alcohol abstinence than did the HC group [OP, Mdn = 1.68; HC, Mdn = 0.45; *U* = 102, *p* = 0.027, *r* = 0.46]. The OP group had a marginally significantly longer duration of nicotine use than did the HC group (OP, *M* = 23.0 (SD = 13.8); HC, *M* = 11.0 (SD = 13.2); *t*(19) = 2.01, *p* = 0.058, *d* = −0.89). The OP group also had a marginally younger age of first tetrahydrocannabinol (THC, a psychoactive component of cannabis) use than did the HC group [OP, Mdn = 15.0; HC, Mdn = 16.5; *U* = 19, *p* = 0.075, *r* = 0.41], and a significantly longer duration of THC use than did the HC group [OP, *M* = 18.3 (SD = 13.4); HC, *M* = 6.00 (SD = 5.62); *t*(17) = −2.82, *p* = 0.012, *d* = −1.06]. The cocaine variable was not compared because the number of HC with a history of cocaine use was too small (*n* = 2).

### 3.3. fMRI task performance and behavioral correlates

#### 3.3.1. fMRI task accuracy and RT performance

**FIGURE 2:**
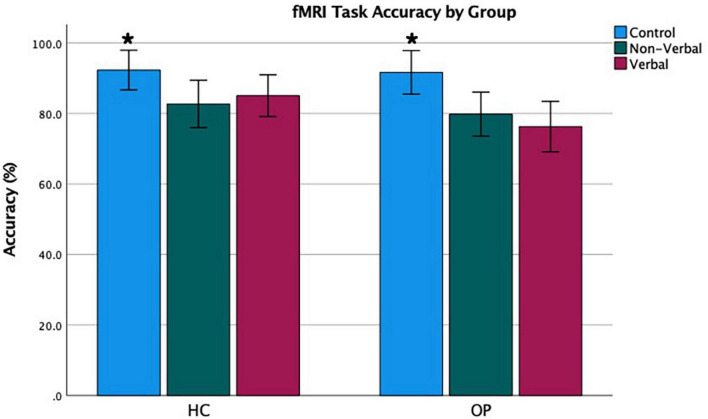
Functional magnetic resonance imaging task accuracy by group. Error bars represent a 95% confidence interval of the true mean. *Accuracy was highest in the control condition.

**FIGURE 3:**
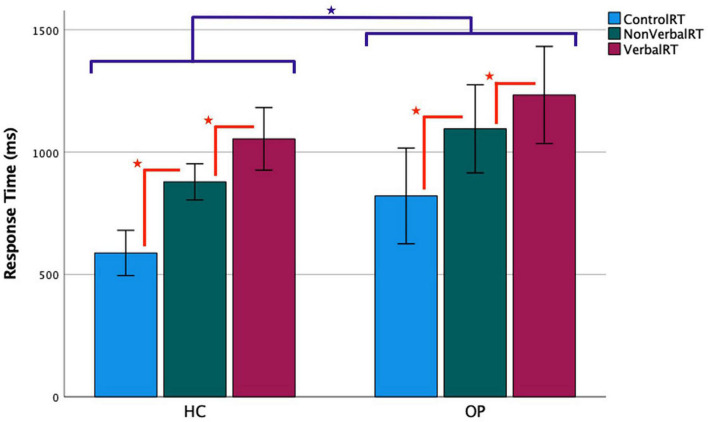
Functional magnetic resonance imaging task response time by group. Accurate trials only were included in the analysis. Error bars represent a 95% confidence interval of the true mean. ^
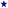
^ = the OP group’s RTs were slower than the HC group’s, *p* < 0.05. ^
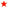
^ = RTs progressively increased from control to non-verbal to verbal conditions, all *p* < 0.05.

Mean accuracy and RTs were computed for the three test conditions (verbal, non-verbal, and control) for each group. Values can be seen in Table 3 and Figures 2, 3. A 3 (condition: verbal, non-verbal, and control) × 2 (group: HC and OP) mixed-design ANOVA yielded a significant main effect of condition on participants’ accuracy (*F*_(2,48)_ = 17.2, *p* < 0.001, η_*p*_^2^ = 0.42). There was neither a main effect of group (*F*_(1,24)_ = 1.59, *p* = 0.219, η_*p*_^2^ = 0.062) nor an interaction condition by group (*F*_(2,48)_ = 1.86, *p* = 0.17, η_*p*_^2^ = 0.072), indicating that the groups did not differ in accuracy performance. *Post hoc* pairwise LSD tests revealed that participants achieved higher accuracy on the control condition (*M* = 92.0, SD = 9.57) than on the verbal (*M* = 80.7, SD = 11.5) and non-verbal (*M* = 81.3, SD = 10.6) conditions.

**TABLE 3:** Behavioral fMRI task performance by group.

	OP (*N* = 13)	HC (*N* = 13)
Control accuracy	91.7 (10.2) [85.5, 97.8], *W* = 0.76, ***p* = 0.002**	92.3 (9.30) [86.7, 97.9], *W* = 0.78, ***p* = 0.004**
Non-verbal accuracy	79.8 (10.3) [73.6, 86.1], *W* = 0.90, *p* = 0.12	82.7 (11.1) [76.0, 89.4], *W* = 0.87, *p* = 0.06
Verbal accuracy	76.3 (11.8) [69.1, 83.4], *W* = 0.96, *p* = 0.78	85.1 (9.82) [79.1, 91.0], *W* = 0.82, ***p* = 0.01**
Control response time	821 (324) [626, 1,017], *W* = 0.94, *p* = 0.44	588 (153) [496, 680], *W* = 0.97, *p* = 0.93
Non-verbal response time	1,095 (298) [915, 1,276], *W* = 0.96, *p* = 0.70	879 (122) [805, 953], *W* = 0.93, *p* = 0.31
Verbal response time	1,234 (328) [1,035, 1,432], *W* = 0.95, *p* = 0.67	1,054 (212) [926, 1,182], *W* = 0.91, *p* = 0.17

Values and statistics are listed as in Tables 1, 2.

The same 3 × 2 mixed-design ANOVA was conducted for the RT data, which yielded a main effect of condition (*F*_(1.38,33.1)_ = 99.0, *p* < 0.001, η_*p*_^2^ = 0.81). There was also a main effect of group (*F*_(1,24)_ = 5.16, *p* = 0.032, η_*p*_^2^ = 0.18), indicating that the OP group took longer than the HC group did to respond overall. There was no significant interaction of condition by group (*F*_(1.38,33.1)_ = 0.374, *p* = 0.61, η_*p*_^2^ = 0.015). To understand the main effect of condition, *post-hoc* pairwise LSD tests compared the participants’ RTs between all condition pairings, all *p* < 0.001. Responses to control trials were fastest (*M* = 704.6, SD = 275.1), followed by non-verbal trials (*M* = 987.1, SD = 249.0), and then verbal trials, which were slowest (*M* = 1,144.1, SD = 285.8).

#### 3.3.2. Behavioral correlates with fMRI task performance

To shed light on the influence of cognitive and behavioral processes on the fMRI task performance, correlations within groups were run between accuracy and RT scores on both WM conditions and the following variables: age, education, BIS, digit span, finger tapping, and the color change visual WM tasks (exploratory and uncorrected for multiple tests).

##### 3.3.2.1. OP group results

In the OP group, correlations were revealed between age and verbal and non-verbal accuracy [verbal: *r*(11) = −0.76, *p* = 0.003; non-verbal: *r*(11) = −0.63, *p* = 0.020] as well as non-verbal RT [*r*(11) = 0.63, *p* = 0.022]. These correlations indicated that older age was associated with lower accuracy and slower RTs. Finger tapping with the dominant hand correlated with verbal [*r*(11) = −0.66, *p* = 0.015] and non-verbal RT [*r*(11) = −0.62, *p* = 0.025], reflecting that slower finger tapping was associated with slower RTs. The digit span sum score marginally correlated with verbal accuracy performance [*r*(11) = 0.55, *p* = 0.053], showing that higher number span was associated with higher verbal WM. Similarly, the color change visual WM task marginally correlated with non-verbal accuracy performance [*r*(11) = 0.52, *p* = 0.070], showing that visual WM capacity was associated with non-verbal WM. Variables of education and BIS did not correlate with either WM condition accuracy or RT performance, all *p*-values > 0.37.

##### 3.3.2.2. HC group results

In the HC group, correlations were revealed between age and verbal RT [*r*(11) = 0.70, *p* = 0.008] and non-verbal RT [*r*(11) = 0.67, *p* = 0.012]. As with the OP group, these correlations indicated that older age was associated with slower RTs. All other comparisons were not significant, all *p*-values > 0.24.

##### 3.3.2.3. Impact of age on fMRI task performance

Because age correlated with accuracy and RT, analyses of covariance (ANCOVAs) were run on both performance measures to find out if the original findings remained when controlling for age. The ANCOVAs revealed no main effect of condition for accuracy (*F*_(2,46)_ = 0.144, *p* = 0.867, η_*p*_^2^ = 0.006) or RT (*F*_(2,46)_ = 2.38, *p* = 0.104, η_*p*_^2^ = 0.094) when age was included in the model. However, group differences remained for RT (i.e., the OP group was slower than the HC group). Based on these results, age likely influenced the condition effects revealed in the original ANOVA. However, because group differences persisted for RT, age did not fully account for the OP group’s slower RTs.

### 3.4. fMRI results

**FIGURE 4:**
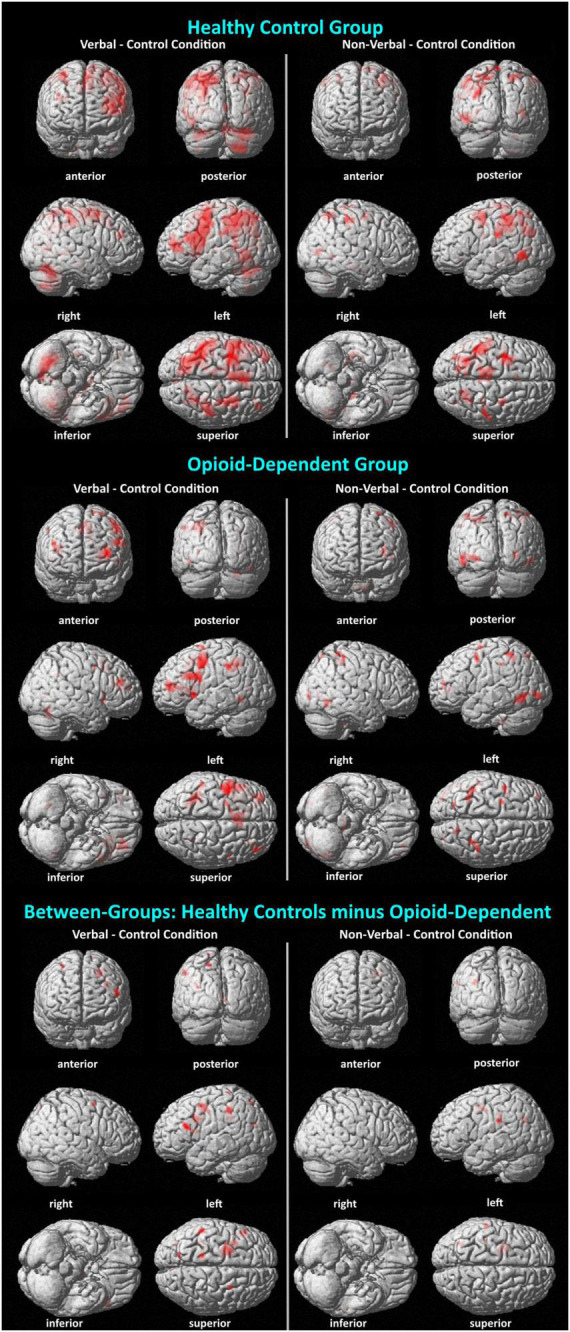
Visualization of BOLD activation associated with verbal and non-verbal WM. Surface renderings of BOLD activations display activations listed in Table 4, as well as activations that did not pass a cluster size threshold of *p* < 0.05. Thus, there are more activations shown here than in Table 4. Minimum cluster size = 10 voxels; all activations *p* < 0.001. In all comparisons, the activations are shown for the WM condition (verbal or non-verbal) minus the control condition. In the between-groups comparison, activations reflect a double subtraction [HC (WM – control conditions) – OP (WM – control conditions)].

Analyses for this study focused on activations during the maintenance phase of the verbal, non-verbal, and control WM tasks. For within-group analyses, fMRI beta weight contrast values were computed. Positive contrast values indicated higher BOLD signal during the verbal or non-verbal WM task compared to the control task (see Table 4 and Figure 4). The results of the verbal WM task in both groups generally agreed with previous findings that included activation with the cerebro-cerebellar circuit and secondary motor regions (Chen and Desmond, 2005; Marvel and Desmond, 2012; Marvel et al., 2012, 2022). Both groups revealed activity in the prefrontal cortex and superior cerebellum. The HC group also activated verbal WM typical regions in the premotor cortex and thalamus. The OP group additionally activated the inferior frontal gyrus (Broca’s area), which is often observed during verbal WM. Results of the non-verbal WM condition in both groups showed a tendency to activate regions related to primary visual and visuomotor processes. HC group activation of the premotor cortex in the non-verbal WM condition was also consistent with previous findings describing a role for secondary motor regions in non-verbal WM (Pollmann and Von Cramon, 2000; Champod and Petrides, 2007; Liao et al., 2014; Sobczak-Edmans et al., 2016; Marvel et al., 2019).

**TABLE 4:** BOLD activation clusters associated with verbal and non-verbal WM.

Cluster size	*t*-score	MNI coordinates (*x*, *y*, *z*)	Brain region (Brodmann area)
**Within-groups analysis by condition**			
**Healthy controls: verbal – control condition**			
2,713	24.72	**24, –64, –32**	Right superior cerebellum, lobe VI
7,385	14.57	**–52, –38, 46**	Left supramarginal gyrus (BA 40)
6,989	12.74	**–28, 0, 50**	Left middle frontal gyrus (premotor cortex, BA 6)
2,925	7.86	30, –12, 52	Right precentral gyrus (premotor cortex, BA 6)
68	7.43	40, −24, –16	Right hippocampus
434	7.02	**–16, –12, 6**	Left thalamus
183	5.88	**36, 38, 24**	Right middle frontal gyrus (dorsolateral prefrontal cortex, BA 9)
173	4.91	**2, –80, 12**	Cuneus (primary visual cortex, BA 17)
**Opioid-dependent: verbal – control condition**			
1,595	7.67	**–46, 20, 26**	Left inferior frontal gyrus (Broca’s area, BA 44)
430	7.40	**10, 18, 44**	Right medial frontal gyrus (frontal eye fields, BA 8)
674	7.21	**–50, –40, 40**	Left supramarginal gyrus (BA 40)
81	7.26	**24, –64, −26**	Right superior cerebellum, lobe VI
88	6.23	**–40, –56, –6**	Left fusiform gyrus (BA 37)
108	6.16	**–48, 12, –2**	Left inferior frontal gyrus (Broca’s area, BA 44)
137	5.15	**32, 32, 22**	Right middle frontal gyrus (dorsolateral prefrontal cortex, BA 9)
**Healthy controls: non-verbal – control condition**			
1,958	10.46	**–32, –66, 30**	Left angular gyrus (BA 39)
525	9.37	**–42, –56, –4**	Left fusiform gyrus (BA 37)
143	8.71	34, –70, 6	Right cuneus (primary visual cortex, BA 17)
606	7.85	–34, –4, 52	Left middle frontal gyrus (premotor cortex, BA 6)
367	7.44	–4, –32, 70	Left precentral gyrus (premotor cortex, BA 6)
262	5.60	22, –62, 58	Right superior parietal lobe (visuomotor coordination, BA 7)
201	5.33	42, –34, 52	Right post central gyrus (primary somatosensory cortex, BA 1)
105	5.18	**32, –6, 54**	Right middle frontal gyrus (premotor cortex, BA 6)
**Opioid-dependent: non-verbal – control condition**			
362	8.38	**–48, –62, –12**	Left fusiform gyrus (BA 37)
172	6.56	**–28, –52, 50**	Left superior parietal lobule (visuomotor coordination, BA 7)
144	6.54	38, –40, 54	Right supramarginal gyrus (BA 40)
84	5.62	**50, –64, –8**	Right fusiform gyrus (BA 37)
**Between-groups analysis by condition (healthy controls minus opioid-dependent)**			
**Verbal – control condition**			
272	5.25	**–24, –2, 48**	Left middle frontal gyrus (premotor cortex, BA 6)
118	5.08	**–34, 8, 32**	Left middle frontal gyrus (frontal eye fields, BA 8)
105	3.84	**–50, 22, 18**	Left inferior frontal gyrus (Broca’s area, BA 44)
**Non-verbal – control condition**			
105	5.37	**–22, –8, 44**	Left middle frontal gyrus (premotor cortex, BA 6)

Clusters were revealed by contrasts of the experimental WM condition (e.g., verbal or non-verbal) minus the control condition during the maintenance phase of the task. All clusters passed a significance threshold of *p* < 0.001, uncorrected, and a cluster size threshold of *p* < 0.05, uncorrected. Reported clusters surpassed a minimum of 10 voxels per cluster. Clusters are listed by decreasing *t*-score of the peak voxel within a cluster. Bolded regions of interest reflect consistency with previous WM imaging studies (Pollmann and Von Cramon, 2000; Chen and Desmond, 2005; Champod and Petrides, 2007; Marvel and Desmond, 2012; Marvel et al., 2012, 2019, 2022; Liao et al., 2014; Sobczak-Edmans et al., 2016). Coordinates are listed according to the Montreal Neurological Institute (MNI) atlas within SPM12. MNI coordinates were converted to Talairach using the MNI2TAL function within the BioImage Suite Web (https://bioimagesuiteweb.github.io/webapp/#), which includes a Brodmann area (BA) overlay, and then cross-referenced against the Talairach atlas (Talairach and Tournoux, 1988). Note that clusters revealed by the within-group contrasts were used for subsequent ROI analyses.

When groups were compared directly, HC–OP yielded differences in the inferior frontal gyrus in the verbal condition and in the left premotor cortex in the verbal and non-verbal conditions. No significant group differences were found for the OP–HC comparison of contrasts for either WM condition.

### 3.5. Region of interest results

#### 3.5.1. ROI correlations with fMRI task performance

**FIGURE 5:**
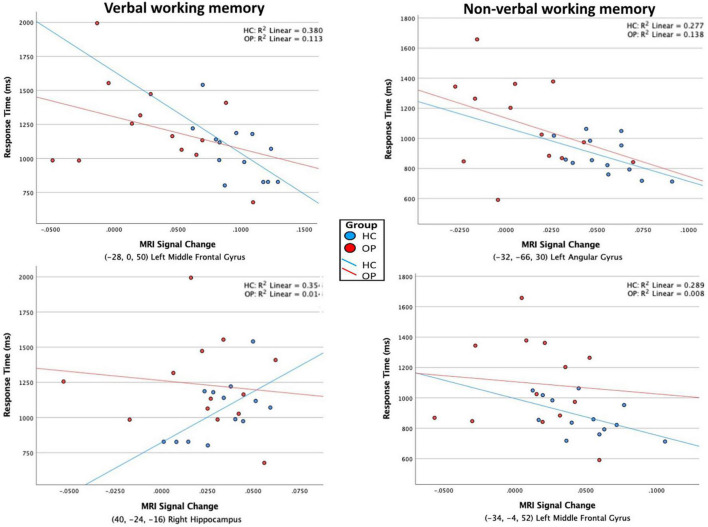
Scatterplots of the association between RT and BOLD MRI signal. ROI clusters were determined by within-group contrasts between verbal – control and non-verbal – control conditions, listed in Table 4. Significant correlations were found only in the HC group, indicated by the individual values and regression lines (blue). In the verbal WM condition **(left column)**, increased activity in the left middle frontal gyrus (premotor cortex, BA 6) was associated with faster RTs, whereas increased activity in the right hippocampus was associated with slower RTs. In the non-verbal WM condition **(right column)**, increased activity in the left angular gyrus (BA 39) and left middle frontal gyrus (premotor cortex, BA6) were associated with faster RTs.

Further analyses explored whether any of the clusters revealed in the within-groups contrasts correlated with task performance. Within the HC group, a negative correlation was found between RT on the verbal WM condition and the BOLD signal in the left middle frontal gyrus (premotor cortex) [*r*(11) = −0.62, *p* = 0.025, cross-reference with Table 4 for coordinates]. A positive correlation was found between RT and BOLD signal in the right hippocampus [*r*(11) = 0.60, *p* = 0.032]. For the non-verbal WM condition in the HC group, there was a marginally significant negative correlation between the RT and BOLD signal in the left angular gyrus [*r*(11) = −0.53, *p* = 0.065] and between the RT and BOLD signal in the left middle frontal gyrus (premotor cortex) [*r*(11) = −0.54, *p* = 0.058]. Scatterplots displaying all four correlations are presented in Figure 5. In the OP group, one marginally significant and negative correlation was found between RT on the verbal WM condition and BOLD signal in the left inferior frontal gyrus [*r*(11) = −0.54, *p* = 0.057], but visual inspection of the scatterplot indicated this relationship was skewed because of one data point. With that data point removed, the correlation was not significant [*r*(10) = −0.16, *p* = 0.59]. No significant correlations were found between ROI BOLD signals and accuracy for either group, all *p*-values > 0.12.

#### 3.5.2. ROI correlations with drug history variables

Significant correlations between drug history variables and the ROIs for each group are reported in Table 5. Both groups showed correlations between ROI BOLD signal and use of alcohol and nicotine. Most correlations were related to the ROI clusters associated with verbal WM. Correlations involving non-verbal ROI clusters were observed in the angular gyrus and superior parietal lobe.

**TABLE 5:** Significant correlations between drug history variables and regions of interest (ROIs).

Drug history variable	Brain region	MNI coordinates (*x*, *y*, *z*)	Statistics
**Healthy controls**			
Age of first alcohol use	Right hippocampus	40, –24, –16 (verbal)	*r*(10) = −0.60, *p* = 0.041
	Cuneus (primary visual cortex, BA 17)	2, –80, 12 (verbal)	*r*(10) = −0.76, *p* = 0.004
Duration of alcohol use	Right hippocampus	40, –24, –16 (verbal)	*r*(10) = 0.77, *p* = 0.003
	Right middle frontal gyrus (dorsolateral prefrontal cortex, BA 9)	36, 38, 24 (verbal)	*r*(10) = 0.67, *p* = 0.016
	Left angular gyrus (BA 39)	–32, –66, 30 (non-verbal)	*r*(10) = −0.71, *p* = 0.010
Age of first nicotine use	Cuneus (primary visual cortex, BA 17)	2, −80, 12 (verbal)	*r*(7) = −0.72, *p* = 0.029
Age of first THC use	Cuneus (primary visual cortex, BA 17)	2, –80, 12 (verbal)	*r*(4) = −0.90, *p* = 0.015
THC abstinence duration	Left angular gyrus (BA 39)	–32, –66, 30 (non-verbal)	*r*(4) = −0.81, *p* = 0.049
**Opioid-dependent**			
Age of first alcohol use	Right medial frontal gyrus (frontal eye fields, BA 8)	10, 18, 44 (verbal)	*r*(9) = 0.61, *p* = 0.045
	Left supramarginal gyrus (BA 40)	–50, –40, 40 (verbal)	*r*(9) = 0.72, *p* = 0.013
	Right superior cerebellum, lobe VI	24, –64, –26 (verbal)	*r*(9) = 0.83, *p* = 0.002
	Left inferior frontal gyrus (Broca’s area, BA 44)	–48, 12, –2 (verbal)	*r*(9) = 0.73, *p* = 0.010
	Left superior parietal lobe (visuomotor coordination, BA 7)	–28, –52, 50 (non-verbal)	*r*(9) = 0.63, *p* = 0.037
Duration of alcohol use	Left inferior frontal gyrus (Broca’s area, BA 44)	–46, 20, 26 (verbal)	*r*(9) = 0.61, *p* = 0.047
Duration of nicotine use	Left inferior frontal gyrus (Broca’s area, BA 44)	–46, 20, 26 (verbal)	*r*(10) = 0.65, *p* = 0.024
Cocaine use frequency	Left fusiform gyrus (BA 37)	–40, –56, –6 (verbal)	*r*(9) = −0.68, *p* = 0.022
Oxycontin abstinence duration	Left fusiform gyrus (BA 37)	–40, –56, –6 (verbal)	*r*(3) = 0.94, *p* = 0.017
Oxycontin use frequency	Right middle frontal gyrus (dorsolateral prefrontal cortex, BA 9)	32, 32, 22 (verbal)	*r*(3) = 0.95, *p* = 0.013

Pearson and Spearman correlation tests were run between the drug history variables in Table 2 and the ROIs from Table 4. Coordinates are listed according to the Montreal Neurological Institute (MNI) atlas within SPM12, followed by the verbal or non-verbal WM condition in which the ROI was revealed. Pearson’s correlations were used for all normally distributed variables and Spearman’s rank correlations were used for all non-normal distributions. All significant correlations *p* < 0.05 are reported below.

## 4. Discussion

In this study, functional MRI was used to investigate the neural correlates of verbal and non-verbal WM in opioid-dependent, methadone-maintained individuals. It was hypothesized that fMRI would reveal neural activation patterns in the opioid-dependent participants that differed from those observed in HC during verbal and non-verbal WM demands. Specifically, it was hypothesized that the opioid-dependent group would express greater neural activity than HC in several previously established brain regions as compensation for cognitive deficits. This hypothesis was not supported. In fact, observations indicated the opposite effect: group differences revealed higher activity in the HC than OP group within the inferior frontal gyrus in the verbal WM condition and in the left premotor cortex in the verbal and non-verbal WM conditions.

A side-by-side comparison of within-group brain activations revealed overlap, such as in the prefrontal cortex, supramarginal gyrus, and superior cerebellum, but each group additionally elicited activity in distinct regions, many of which were expected for a Sternberg WM task. For example, the HC group showed verbal WM activity in the thalamus and cuneus that was not observed in the OP group. Conversely, the OP group showed verbal WM activity in the fusiform gyrus that was not observed in the HC group (Note that left inferior frontal gyrus activity was not observed as an individual cluster in the HC group, as expected and observed in the OP group, but in the HC group this region was likely folded into the activation cluster observed in the left middle frontal gyrus that was 6989 voxels. This drove the between-group difference for greater HC than OP activity in the inferior frontal gyrus). The neural correlates of non-verbal WM have been examined to a lesser degree in the literature. However, our data showed consistency with prior reports in both groups for eliciting the fusiform gyrus. The HC group additionally recruited the right middle frontal gyrus (right premotor cortex) and angular gyrus during non-verbal WM. Taken together, these findings support consistencies with the existing literature of activations.

There were also several brain activations in regions that differed from the existing literature for Sternberg-elicited activity in verbal and non-verbal WM, as indicated (non-bolded) in Table 4. For instance, activity in the right precentral gyrus (premotor cortex) and right hippocampus were observed in the HC group during verbal WM, regions that are not typically associated with Sternberg tasks of verbal WM. The right precentral gyrus may have been recruited by controls as part a motor strategy for rehearsing verbal stimuli (Marvel et al., 2019). Meanwhile, recent research has suggested that the hippocampus can be recruited during more challenging WM tasks (Yonelinas, 2013; Ezzati et al., 2016; Kolarik et al., 2016; Kessels and Bergmann, 2022). Increased activity in both regions may have reflected an adaptive response to rehearse verbal stimuli under high WM demands.

Because the literature on the neural correlates of non-verbal WM is not as extensive as that of verbal WM, the novel regions observed in this study are of much interest. In the HC group, activations were observed in the right cuneus, left middle frontal and left precentral gyrus (both including premotor cortex), right postcentral gyrus (primary somatosensory cortex), and right superior parietal lobe. Recruitment of these regions within the current task, however, is reasonable. The cuneus plays a role mainly in visual processing, yet recent research has also reported a role in WM, possibly underlying a visual contribution to the rehearsal of non-verbal stimuli (Vanni et al., 2001; Owens et al., 2018; Hallenbeck et al., 2021). Activity within the left middle frontal gyrus and left precentral gyrus are consistent with what has been observed in verbal WM, as well as the notion that recruiting motor-related regions support motor strategies for rehearsing non-verbalizable symbols (Liao et al., 2014; Marvel et al., 2019). The right postcentral gyrus, which comprises the primary somatosensory cortex, has connections with motor areas in addition to its main role in processing sensory information, possibly contributing further to motor strategies as a process of non-verbal WM (Lee et al., 2013; Kropf et al., 2019). Finally, the right superior parietal lobe plays a role in visuospatial and attentional processing, along with the manipulation of information in WM, consistent with processing the symbolic nature of the non-verbal stimuli (Wager and Smith, 2003; Koenigs et al., 2009). In the OP group, novel non-verbal WM activity was observed in the right supramarginal gyrus. However, the supramarginal gyrus is normally involved in phonological processing and maintenance of verbal WM (Salmon et al., 1996; Mckenna et al., 2013; Deschamps et al., 2014).

Taken together, the pattern of activations observed for verbal and non-verbal WM for the participants were either consistent with the literature or, when novel, made sense given what is known about the regions engaged. The primary activation differences between the groups may reflect inability by the OP group to engage “typical” strategies and/or differences in the approach used to complete the task. For example, increased left premotor and inferior frontal cortical activity by the HC group versus OP group during verbal WM may represent a weaker ability or tendency in the OP group to engage common nodes within the verbal WM circuit that is underlies rehearsal. The unusual activity in the right supramarginal gyrus observed during non-verbal WM in the OP group could represent this group’s strategy to verbalize non-verbal stimuli rather than engage more common motor-related rehearsal strategies.

Behavioral performance between the groups was equated for accuracy; however, RTs were slower overall in the OP group. Given that the groups were equated for age, and group differences remained when age was accounted for, a difference in RT specifically suggests that the OP group’s performance strategies were ultimately successful but perhaps less efficient than those of the HC group. The slower, yet accurate response time, is in line with prior findings from Marvel et al. (2012), which found that methadone-maintained participants had a slower response time and exhibited a different pattern of activity than did HC during a verbal WM task, while maintaining similar accuracy.

By measuring MRI signal within ROIs, it was found that several brain regions correlated with RTs in the HC. Namely, in the verbal WM condition, increased activity in the left premotor cortex correlated with faster RTs. This region may play a role for inner speech and verbal WM rehearsal, which, when engaged, could have facilitated performance in the current task (Marvel and Desmond, 2012; Marvel et al., 2019). By contrast, increased activity in the right hippocampus was associated with slower RTs. While hippocampal activity is not commonly observed during verbal WM, it has been found to engage in WM under more challenging WM conditions (Yonelinas, 2013; Ezzati et al., 2016; Kolarik et al., 2016; Kessels and Bergmann, 2022). It is possible that increased engagement of this region during the maintenance phase reflected higher difficulty to complete the task by some HC participants, and this ultimately slowed decision making (RTs). In the non-verbal WM condition, increased activity in the premotor region and left angular gyrus (typically involved in verbal processes, Seghier, 2013) were associated with slower RTs, potentially reflecting less efficient (and perhaps verbal-related) rehearsal strategies by controls. No associations between the ROIs and RTs were observed in the OP group, which is notable given that the OP group had slower RTs.

Performance on the fMRI WM tasks was found to correlate with demographic variables and cognitive tests scores, indicating that these factors differentially influenced each group’s performance. For example, the education level in the OP group was significantly lower than that of the HC group. This difference may explain why the OP group scored lower on the digit span and color change visual WM tasks than did the HC group, both cognitive tests for WM performance. However, education level did not correlate with fMRI task performance in either group, implying that education level was not the primary contributor to RT group differences. The OP group scored higher on the BIS than did the HC group, indicating that the OP participants were more impulsive overall. Like education level, however, impulsivity (BIS) did not show a relationship with fMRI task performance. Both groups were affected by age, such that RTs slowed with age. In the OP group only, there were additional associations. Older age was associated with lower WM accuracy. Slower finger tapping was associated with slower RTs. Although marginally significant, it was relevant to note that performance on the digit span task, which required verbal recall of numbers, correlated with verbal WM accuracy. Conversely, performance on the color change visual WM task, which required encoding, retention, and recognition of a spatial array of colored symbols, marginally correlated with non-verbal WM accuracy. Thus, it appeared that the OP group’s performance was influenced by age, general motor function, and domain-specific WM capacity.

The two groups also differed in their drug histories. Alcohol and THC use were the biggest differences between the OP and HC groups. The OP group reported significantly younger ages of first alcohol and THC use, and a longer duration of THC use over their lifetimes, relative to that of the HC group. However, all 13 OP participants had a history of THC use while only six HC participants had used THC. There was some recreational cocaine use in the HC group by two participants. Only the OP group reported any opioid use. Drug history variables provided additional insights into the activations observed in the OP group. For instance, younger age of first alcohol use correlated with lower activation in the frontal eye fields, left supramarginal gyrus, right superior cerebellum, left inferior frontal gyrus, and left superior parietal lobule—regions that have been noted as relevant to verbal and non-verbal WM.

### 4.1. Limitations

This study had several limitations that should be considered when interpreting the data. A primary limitation is the small sample size. This study included a carefully selected group of participants with a history of opioid addiction who were receiving methadone treatment and did not have a history of mood or psychotic disorders or alcoholism, all of which are commonly observed in substance-abusing populations. If the sample had included people with such psychiatric histories, however, this could have confounded the results and rendered the data difficult to interpret. Nonetheless, the sample size was not large enough to use a more stringent threshold of family wise error rate (FWE) <0.05 in the fMRI data, and our correlations between variables were exploratory and uncorrected; they should be considered preliminary until future studies can be conducted on larger sample sizes. A second limitation of this study was that results may be confounded by the administration of methadone to the OP group. Although it was intentional to include OP participants who were undergoing treatment to obtain a somewhat homogeneous sample with well-documented clinical histories (aspects that are favorable to research), methadone can have its own effects on WM. For example, Mintzer et al. (2005), Verdejo et al. (2005), and Darke et al. (2012) found that WM was most impaired for methadone-maintained patients as compared to abstinent ex-abusers and HC. To mitigate the immediate effects of methadone on cognition, participants withheld their morning dose until after the MRI was completed. Because participants routinely took their dose each morning at the clinic, abstinence was roughly 20−24 h *post* methadone dose, which is well past methadone’s peak effects on cognition and physiology, and represents a “trough” period when methadone’s effect return to baseline (Walsh et al., 1994; Eissenberg et al., 1999). A withdrawal scale (COWS) was administered after the MRI to measure withdrawal effects, which were minimal to absent for all participants. Nonetheless, the effects of methadone on cognition or brain physiology cannot be ruled out here. A third limitation of this study is that the overdose history of the opioid-dependent group was unknown. Overdosing on opioids, such as heroin, can result in brain damage through hypoxia, a lack of oxygen flow to the brain and subsequently contributes to many cognitive impairments, including WM (Anderson and Arciniegas, 2010; Corrigan and Adams, 2019). Some participants may have had more extreme or numerous overdoses than others, resulting in differences in brain structure and functions. This variable could not be controlled for in the data analyses. A fourth limitation is related to endogenous peptide levels that can be altered in people who abuse opioids, which has the potential to impact cognitive abilities (Shahkarami et al., 2019). Plasma measures of endogenous opioid peptides were not obtained, and therefore, group differences were not considered in the analyses (Trigo et al., 2010; Toubia and Khalife, 2019). A fifth limitation was that groups had unequal education levels. Even though education was not correlated with the fMRI task performance, the small sample size limited statistical power to fully explore education’s effects on the current data. Finally, a sixth limitation was that information was not collected regarding whether participants were multilingual. Research has suggested that multilingual individuals have greater executive functioning capabilities than do monolingual individuals, which could have influenced findings in the current study (Quinteros Baumgart and Billick, 2018). However, because each study group would have been equally likely to contain multilingual participants, any such effects would have been controlled for in the data.

## 5. Summary

In this study, fMRI was used to investigate the neural correlates of verbal and non-verbal WM in opioid-dependent, methadone-maintained individuals. Brain activity consistent with previous research was observed within both the OP and HC groups, but clear differences in activations were also observed between the two groups. Differences in brain activations were likely related to differences in cognitive strategies. Slower response times for the OP group were possibly impacted by early exposure to illicit substances. Continued research into understanding the factors that contribute most to substance use, and how substance use impacts the brain, cognition, and behaviors, is warranted to most effectively treat those with substance use disorders.

## Data availability statement

The raw data supporting the conclusions of this article will be made available by the authors, without undue reservation.

## Ethics statement

The studies involving human participants were reviewed and approved by the Johns Hopkins Institutional Review Board. The patients/participants provided their written informed consent to participate in this study.

## Author contributions

CM contributed to the study conception, design, data collection, and data curation. JB, PN, and CM contributed to the data analyses and the first draft of the manuscript. All authors contributed to manuscript revision, read, and approved the submitted version.
